# Comment on “Hypertension, pregnancy, and weather: is seasonality involved?”

**DOI:** 10.1590/1806-9282.20251974

**Published:** 2026-08-10

**Authors:** Xue Wei

**Affiliations:** 1 Taizhou Meteorological Bureau – Taizhou, China.

Dear Editor,

Recently, I read a research article titled “Hypertension, pregnancy and weather: is seasonality involved?”^1^ and I have some viewpoints to share with you and the readers.

Pregnancy-induced hypertension is a disease caused by both internal and external factors^2,3^. Self-factors include genetic susceptibility, smoking, mental health, basic diseases, etc., while external factors include living environment, health conditions, climate factors, etc. At present, the academic community believes that extreme supercooling or overheating temperature will lead to an increase in the incidence of pregnancy-induced hypertension. The author of this article only observed and listed the two climate measurable indicators of local temperature and humidity for descriptive analysis, while ignoring the consistency of the baseline and other external factors of the overall research object.

The study samples under different conditions need to be grouped according to different characteristics after homogeneity analysis. Comparative analysis between subgroups seems to yield more reliable results.

On the other hand, according to the basic understanding of meteorology, the local climate characteristics are basically consistent over a short period of several decades. If the study of temperature and humidity changes may increase the incidence of pregnancy-induced hypertension, we need to find a scientific external control. Other climatic factors that the external control can meet the requirements of degassing temperature and humidity are basically the same as those of the study group, and the characteristics of the study sample are basically the same.

## Data Availability

The datasets generated and/or analyzed during the current study are available from the corresponding author upon reasonable request.
